# Practice effects in healthy adults: *A longitudinal study on frequent repetitive cognitive testing*

**DOI:** 10.1186/1471-2202-11-118

**Published:** 2010-09-16

**Authors:** Claudia Bartels, Martin Wegrzyn, Anne Wiedl, Verena Ackermann, Hannelore Ehrenreich

**Affiliations:** 1 Division of Clinical Neuroscience, Max Planck Institute of Experimental Medicine, Göttingen, Germany

## Abstract

**Background:**

Cognitive deterioration is a core symptom of many neuropsychiatric disorders and target of increasing significance for novel treatment strategies. Hence, its reliable capture in long-term follow-up studies is prerequisite for recording the natural course of diseases and for estimating potential benefits of therapeutic interventions. Since repeated neuropsychological testing is required for respective longitudinal study designs, occurrence, time pattern and magnitude of practice effects on cognition have to be understood first under healthy good-performance conditions to enable design optimization and result interpretation in disease trials.

**Methods:**

Healthy adults (N = 36; 47.3 ± 12.0 years; mean IQ 127.0 ± 14.1; 58% males) completed 7 testing sessions, distributed asymmetrically from high to low frequency, over 1 year (baseline, weeks 2-3, 6, 9, months 3, 6, 12). The neuropsychological test battery covered 6 major cognitive domains by several well-established tests each.

**Results:**

Most tests exhibited a similar pattern upon repetition: (1) Clinically relevant practice effects during high-frequency testing until month 3 (Cohen's *d *0.36-1.19), most pronounced early on, and (2) a performance plateau thereafter upon low-frequency testing. Few tests were non-susceptible to practice or limited by ceiling effects. Influence of confounding variables (age, IQ, personality) was minor.

**Conclusions:**

Practice effects are prominent particularly in the early phase of high-frequency repetitive cognitive testing of healthy well-performing subjects. An optimal combination and timing of tests, as extractable from this study, will aid in controlling their impact. Moreover, normative data for serial testing may now be collected to assess normal learning curves as important comparative readout of pathological cognitive processes.

## Background

Cognitive decline is a common feature of many neuropsychiatric diseases and among the strongest determinants of real-world functioning and quality of life in affected individuals [e.g. [1-4]]. Moreover, it poses enormous and ever-increasing costs on the progressively aging industrial societies.

Efficient treatment of cognitive impairment is urgently needed but not yet available. Therapeutically addressing cognitive outcome requires careful assessment based on comprehensive neuropsychological examination of relevant cognitive domains. Cognitive tests can be applied cross-sectionally to obtain first diagnostic information but solid clinical judgments as well as research require longitudinal observation. In clinical neuropsychology, serial test administration is essential for (1) monitoring of disease progression and/or potential recovery, or (2) evaluating efficacy of a therapeutic agent or other interventions (e.g. rehabilitation programs) in both, randomized clinical trials or clinical follow-up of single cases. Dependent on the underlying questions, testing frequencies have to be adapted to enable measurement of short-term or long-term processes. With repeated testing, however, the phenomenon of 'practice effects', reflecting the capability of an individual to learn and adjust, represents not only an additional important cognitive readout but also an interfering variable complicating result interpretation [5-7].

Practice effects are defined as increase in a subject's test score from one administration to the next in the absence of any interventions. Various reasons have been discussed to explain practice-induced score gains, such as reduced anxiety in or growing familiarity with the testing environment, recall effects, improvement of underlying functions, procedural learning, test sophistication, or regression to the mean [5,8-10]. Furthermore, practice effects seem to be influenced by test (complexity, modality, alternate forms introduced to make them 'repeatable') [9-17], or test-taker characteristics (IQ, age, personality, mood, motivation) [5,6,13,16,18-24], as well as intertrial interval [11,19,25]. Surprisingly, for most cognitive instruments, only test-retest reliability coefficients, usually including not more than 2 sessions, are available. Even the useful concept of the 'reliable change index' corrected for practice and its extensions [e.g. [26-29]] has not been systematically integrated in most test manuals due to the low repetition rate of tests.

If not properly integrated into interpretation of cognitive results, practice effects can easily lead to false conclusions: (1) degenerative processes obscured by practice may be underestimated [30] or (2) treatment effects might be overestimated, particularly in the absence of adequate control groups [31]. Even though the integration of appropriate control groups into clinical treatment trials remains inevitable, a solid prediction of expected practice effects under healthy good-performance conditions is deemed essential for accurate effect size estimation and selection of a suitable test set. In a single case follow-up, this may actually be the only reasonable basis of judgment. Therefore, it is surprising that despite a number of pivotal prior studies dealing with practice effects, comprehensive characteristics of normal performance over time are not available. Specifically, the impact of practice effects on frequent repetitive neuropsychological testing over as long as 1 year, comprising all major cognitive domains, has not been systematically studied in healthy well-performing individuals.

The first objective of the present study has been to start filling this gap by exploring test-specific practice effects on performance in the 6 major cognitive domains upon repetitive testing of healthy subjects over a whole year. The design of intertest intervals has been chosen to meet typical requirements of neuroprotective trials, with short-term high-frequency followed by long-term low-frequency testing. As the second objective, recommendations should be extractable from the findings of the present work to design an optimal neuropsychological testing procedure for future longitudinal clinical research or routine. Finally, the third objective has been to provide the ground for future collection of normative data on longitudinal learning curves as an important but thus far largely ignored diagnostic readout of cognitive abilities.

## Methods

### Participants

The present study was approved by the local ethical committee (Ethikkommission der Medizinischen Fakultät der Georg-August-Universität Göttingen). All study participants gave written informed consent after complete description of the study. Native German speaking healthy subjects were recruited via public advertising and financially compensated upon completion of all follow-up sessions. (In our experience, financial compensation increases motivation of subjects to keep the appointments but is highly unlikely to influence cognitive performance itself.) A total of 36 healthy individuals (21 males and 15 females) with a mean age of 47.3 ± 12.0 years (range 24-69 years) at study entry participated. Prior to enrolment, a standardized, semi-structured interview and a physical screening examination confirmed that subjects were free of significant medical conditions or neuropsychiatric diseases (past or current). Psychopathological ratings (Hamilton Rating Scale for Depression, HAMD [32], mean 2.1 ± 2.9; Positive and Negative Syndrome Scale, PANSS [33], mean 32.3 ± 3.5) further ascertained a healthy sample with an overall high intellectual level of performance as underlined by premorbid (mean 124.2 ± 12.8) and status intelligence (mean 127.0 ± 14.1) quotients. Additionally, a personality questionnaire (revised NEO Personality Inventory, NEO-PI-R [34]) yielded results in the middle normal range (mean *T values *for personality factors: Neuroticism 46.3 ± 9.3; Extraversion 51.7 ± 10.7; Openness 52.2 ± 9.3; Agreeableness 53.4 ± 8.3; Conscientiousness 50.6 ± 8.3). Exclusion criteria comprised use of illicit and prescribed psychoactive drugs as well as nicotine. Urine drug screening (testing for ethanol, cannabinoids, benzodiazepines and cocaine) at baseline and unheralded repeat screens at random intervals verified the drug-free state of all individuals.

### Study design

**Figure 1:**
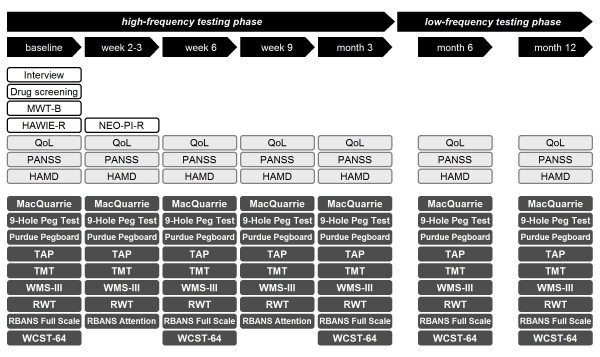
**Study design comprising a comprehensive cross-sectional baseline evaluation and 2 longitudinal phases with high-frequency followed by low-frequency testing**. With a semi-structured interview, sociodemographic variables and medical history were collected. Drug screening: Urine samples of all subjects were tested for ethanol, cannabinoids, benzodiazepines and cocaine at baseline, and tests were repeated randomly afterwards. MWT-B, Mehrfachwahl-Wortschatz-Intelligenz-Test (premorbid intelligence measure); HAWIE-R, revised German version of the Wechsler Adult Intelligence Scale (subtests Information, Similarities, Picture Completion, Block Design); NEO-PI-R, revised NEO Personality Inventory; QoL, quality of life visual-analogue scale; PANSS, Positive and Negative Syndrome Scale; HAMD, Hamilton Rating Scale for Depression; MacQuarrie; MacQuarrie Tapping and Dotting tests; Purdue Pegboard, Purdue Pegboard Test, TAP, Test for Attentional Performance (subtests Alertness, Visual Scanning, Working Memory, Flexibility); TMT, Trail Making Test (A and B); WMS-III, Wechsler Memory Scale - 3^rd ^edition (subtest Letter Number Sequencing); RWT, Regensburger Wortflüssigkeitstest (subtest phonemic verbal fluency); RBANS, Repeatable Battery for the Assessment of Neuropsychological Status (Full Scale, complete RBANS performed; Attention, RBANS Attention subtests only); WCST-64, Wisconsin Card Sorting Test - 64 Card Version.

All screened and included subjects underwent comprehensive neuropsychological and psychopathological testings of approximately 2h duration under standardized conditions (fixed sequence, fixed day time per subject) on 7 occasions in total. The entire study was performed by 2 examiners (trained psychologists). Tests were administered according to standard instructions and, if available, alternate forms were used (for overview see Additional file 1). The longitudinal study design comprised a short-term high-frequency testing phase with a 3-week intertest interval (baseline, week 2-3, week 6, week 9 and month 3) and a long-term low-frequency testing phase (months 6 and 12), amounting to a total duration of 1 year per individual (Figure 1). The rationale of this testing schedule is derived from neuroprotective treatment trials on cognitive outcomes [e.g. [35]]. All included subjects completed all 7 testing sessions as scheduled (no drop outs), resulting in a complete data set without any missing data.

### Neuropsychological test battery

A total of 25 tests were selected to cover major cognitive domains: **(1) attention**: Trail Making Test - part A (TMT A [36]), Repeatable Battery for the Assessment of Neuropsychological Status (RBANS [37]) subtests Coding and Digit Span, Test for Attentional Performance (TAP [38]) subtests Visual Scanning and Alertness; **(2) learning and memory**: RBANS subtests Figure Recall, List Learning, List Recall, List Recognition, Story Memory, Story Recall; **(3) executive functions**: Trail Making Test - part B (TMT B [36]), Wisconsin Card Sorting Test - 64 Card Version (WCST-64 [39]), TAP subtests Flexibility and Working Memory, Wechsler Memory Scale - 3^rd ^edition (WMS-III [40]) subtest Letter Number Sequencing, Regensburger Wortschatztest (RWT [41]) subtest phonemic verbal fluency; **(4) motor functions**: 9-Hole Peg Test [42], Purdue Pegboard Test [43], MacQuarrie Test for Mechanical Ability [44] subtests Tapping and Dotting; **(5) language**: RBANS subtests Picture Naming and Semantic Fluency; and **(6) visuospatial functions**: RBANS subtests Lines and Figure Copy. All listed tests are well-established and have been described in detail as referenced (for a short description see Additional file 1). Of all tests, only the most relevant parameter is presented to avoid overrepresentation of one test. To minimize expected strong recall effects, RBANS short- and long-term memory tests, visuospatial and language functions as well as the WCST-64 have been performed less frequently (baseline, week 6, months 3, 6, 12). Intelligence [premorbid intelligence (Mehrfachwahl-Wortschatz-Intelligenz-Test, MWT-B [45]) and state intelligence (revised German version of the Wechsler Adult Intelligence Scale, short version, HAWIE-R [46])] - as well as personality (NEO-PI-R) measures were performed only at baseline to explore their potential influence on the course of cognitive performance. Current psychopathological symptoms (HAMD, PANSS [32,33]) and quality of life (visual analogue scale ranging from 0-10) were assessed at each testing time-point (Figure 1) to control for their potentially fluctuating nature.

### Statistical analysis

All numerical results are presented as mean ± SD in text/table and mean ± SEM in figures. Statistical tests were two-tailed for all analyses with a conservative significance level at p < 0.01 to account for multiple statistical testing (p < 0.05 was considered only marginally significant). Analysis of variance (ANOVA) for repeated measures with time as independent variable was applied to all individual cognitive tests in order to investigate significance of score changes over time (practice effects). For analysis of cognitive domains, data of all single cognitive tests (always expressed as % individual baseline of the respective test) were combined to yield respective super-ordinate cognitive mean curves. Two sets of ANOVAs were calculated examining performance changes during (1) the high-frequency testing phase, including all testing time-points from baseline to month 3; and (2) the low-frequency testing phase, including all testing time-points from month 3 to 12. Only if differences over time reached significance, effect sizes (Cohen's *d *[47]) were calculated as *d *= (μ_time-point n_-μ_baseline_)/σ_pooled _for determination of the clinical relevance of practice effects. Thus, improvement in speed tests (i.e. reduction in reaction time) or reduction in error rate (WCST-64) resulted in negative effect sizes. For consistency, these negative effect sizes were inverted to express enhanced test performance as positive Cohen's *d*. Altogether, *d *= 0.20-0.49 was considered to represent small, *d *= 0.50-0.79 moderate and *d *≥ 0.80 large effects [47]. To detect potential confounders of cognitive performance, Pearson's correlations were calculated between age, IQ (MWT-B or HAWIE-R IQ), all 5 NEO-PI-R personality factors, HAMD, PANSS total scores and single cognitive measures at baseline. The influence on the course of cognitive performance was further explored using repeated-measures analyses of covariance (ANCOVA) with time as independent variable (baseline to month 12), and the respective potential confounder as covariate. All analyses (Pearson's correlations, repeated-measures ANOVAs and ANCOVAs) were carried out using SPSS 17.0.

## Results

### Significance and clinical relevance of practice effects by test and cognitive domain: main effect of time in repeated-measures ANOVA and Cohen's *d*

Descriptive statistics for all cognitive tests at all 7 time-points are presented in Table 1. Expectedly, repeated-measures ANOVA across the first 5 sessions of the high-frequency testing phase revealed highly significant score increases over time in the vast majority of tests (practice effects in 17 of 25 tests; all p ≤ 0.006), with the exception of TAP Alertness and Working Memory, RBANS subtests Digit Span, List Recognition, Semantic Fluency, Lines, Figure Copy, and MacQuarrie Dotting. Even after a most conservative Bonferroni correction for 25 comparisons, resulting in an adjusted alpha of 0.002, the significance of results is preserved, with the exception of TMT A and WCST-64 (both p = 0.006).

**Table 1:** Neuropsychological follow-up data (N = 36)

						ANOVA for repeated measures													
	
	Baseline		Week 2-3		Week 6		Week 9		Month 3					Month 6		Month 12			
							
Test parameter	Mean	SD	Mean	SD	Mean	SD	Mean	SD	Mean	SD	***F***_***4.32***_^***§***^	***p***_***1***_	*d*	Mean	SD	Mean	SD	***F***_***2.34***_	***p***_***2***_
***ATTENTION***													***0.69***^**‡**^						

TAP Visual Scanning - critical trials	2401.0	604.8	2041.4	550.1	1908.2	519.9	1782.3	519.4	1712.0	550.1	14.64	***<.001***	***1.19***	1782.3	555.8	1728.8	548.6	*2.57*	.*09*

Trail Making Test - part A	29.3	10.7	24.9	9.0	24.5	8.9	22.8	8.6	23.3	12.0	4.43	**.*006***	***0.53***	22.8	8.2	23.4	7.0	*0.44*	.*65*

RBANS Coding	51.6	9.6	56.0	10.6	52.2	9.0	54.5	9.7	55.1	10.0	9.74	***<.001***	***0.36***	54.8	10.3	54.9	8.8	*0.05*	.*95*

TAP Alertness - with cue sound	265.8	44.8	262.8	60.3	261.9	54.8	258.8	65.9	248.9	64.8	*2.39*	.*07*	-	254.4	46.7	264.0	70.3	*1.89*	.*17*

RBANS Digit Span	10.8	2.1	10.3	1.8	10.9	1.9	10.8	2.4	10.7	2.0	*1.00*	.*42*	-	11.1	2.2	11.3	1.9	*2.27*	.*12*

***LEARNING AND MEMORY***													***0.67***^**‡**^						

RBANS Figure Recall	16.6	2.9	-	-	18.4	2.1	-	-	18.4	1.8	*11.65*	***<.001***	***0.75***	18.8	1.3	18.6	1.6	*0.57*	.*57*

RBANS List Recall	7.1	2.2	-	-	7.9	2.0	-	-	8.4	1.6	*13.28*	***<.001***	***0.68***	8.9	1.4	8.4	1.7	*2.33*	.*11*

RBANS List Learning	31.3	3.6	-	-	32.7	4.5	-	-	33.8	3.7	*9.05*	**.*001***	***0.68***	34.8	3.6	33.7	3.7	*2.05*	.*14*

RBANS Story Memory	18.1	4.1	-	-	18.3	3.5	-	-	20.5	2.8	*16.74*	***<.001***	***0.68***	19.8	3.3	19.9	3.7	*1.56*	.*22*

RBANS Story Recall	9.6	1.9	-	-	9.2	1.7	-	-	10.5	1.4	*15.79*	***<.001***	***0.54***	9.9	1.9	10.4	1.6	*2.50*	.*10*

RBANS List Recognition	19.8	0.5	-	-	19.7	0.6	-	-	19.7	0.8	*0.54*	.*59*	-	19.7	0.6	19.7	0.7	*0.07*	.*93*

***EXECUTIVE FUNCTIONS***													***0.50***^**‡**^						

Trail Making Test - part B	70.1	28.5	61.4	29.9	59.7	29.1	52.5	19.5	50.6	22.9	*11.68*	***<.001***	***0.75***	54.8	26.9	53.7	23.7	*0.88*	.*42*

WCST-64 - perseverative errors	9.2	4.8	-	-	7.4	5.0	-	-	6.6	4.2	*6.00*	**.*006***	***0.58***	6.3	4.8	7.2	5.3	*0.59*	.*56*

TAP Flexibility	858.9	320.8	783.6	351.8	750.3	325.8	698.0	332.9	697.9	329.9	*7.46*	***<.001***	***0.49***	685.7	345.7	682.0	305.6	*0.32*	.*73*

WMS-III Letter Number Sequencing	11.4	2.5	12.3	2.3	12.2	2.3	12.6	2.8	12.7	3.0	*6.30*	**.*001***	***0.47***	12.9	2.7	12.9	2.9	*0.19*	.*83*

RWT phonemic verbal fluency	53.6	14.0	60.0	18.0	60.3	16.6	62.8	15.2	56.4	16.1	*10.55*	***<.001***	0.19*	61.2	18.2	63.8	18.3	*16.38*	***<.001***

TAP Working Memory	568.3	160.4	547.7	144.7	569.6	155.7	565.6	176.5	533.0	150.4	*1.55*	.*21*	-	528.3	161.0	533.3	139.3	*0.06*	.*94*

***MOTOR FUNCTIONS***													***0.56***^**‡**^						

9-Hole Peg Test - dominant hand	18.1	2.5	17.4	2.1	16.8	2.4	16.7	2.2	16.5	2.4	*7.11*	***<.001***	***0.65***	16.4	2.3	16.2	2.0	*1.29*	.*29*

Purdue Pegboard Test - assembly	32.4	8.2	33.6	8.5	34.1	8.0	35.0	8.4	36.7	8.3	*8.82*	***<.001***	***0.52***	35.8	9.2	35.5	9.4	*1.70*	.*20*

MacQuarrie Tapping	39.2	7.5	41.4	7.0	41.5	7.8	43.0	8.2	43.1	7.7	*13.08*	***<.001***	***0.51***	42.5	8.2	42.3	7.7	*1.93*	.*16*

MacQuarrie Dotting	66.3	12.4	70.0	12.1	69.8	12.3	70.1	11.1	71.2	10.3	*2.21*	.*09*	-	71.2	12.0	69.5	12.7	*1.86*	.*17*

***LANGUAGE***													***0.00***^**‡**^						

RBANS Picture Naming	10.0	0.0	-	-	9.7	0.5	-	-	10.0	0.2	*7.76*	**.*002***	0.00*	9.8	0.4	10.0	0.0	*4.86*	**.*01***

RBANS Semantic Fluency	23.2	5.2	-	-	25.0	4.8	-	-	24.4	4.5	*2.41*	.*11*	-	25.0	5.2	23.5	5.4	*1.62*	.*21*

***VISUOSPATIAL FUNCTIONS***													-^**‡**^						

RBANS Lines	18.1	2.8	-	-	18.5	2.0	-	-	18.7	1.5	*1.00*	.*38*	-	18.6	1.4	19.0	1.8	*1.23*	.*30*

RBANS Figure Copy	19.9	0.4	-	-	19.9	0.4	-	-	19.9	0.2	*0.50*	.*61*	-	19.9	0.4	19.8	0.4	*1.33*	.*28*

Significant ANOVA (analysis of variance) time effects bolded. ^§^In case of only 3 testings until month 3, df = 2.34. *p*_*1 *_displays significance from baseline to month 3; *p*_*2 *_denotes significance from month 3 to month 12. Cohen's *d *= (μ_month 3 _- μ_baseline_)/σ_pooled _with *d *= 0.20-0.49 denoting small, *d *= 0.50-0.79 moderate, and *d *≥ 0.80 large effect sizes. *indicates *d *< 0.20, i.e. "no effect". Effect sizes presented only if respective differences over time are significant (p < 0.01). ^‡^Cohen's *d *for cognitive domains expressed as mean of single-test effect sizes of the respective cognitive domain.

TAP, Test for Attentional Performance; RBANS, Repeatable Battery for the Assessment of Neuropsychological Status; WCST-64, Wisconsin Card Sorting Test - 64 Card Version; WMS-III, Wechsler Memory Scale - 3^rd ^edition; RWT, Regensburger Wortflüssigkeitstest.

To estimate the magnitude of the observed significant practice effects, most prominent from baseline to month 3, the Cohen's *d *statistic of each test and cognitive domain was calculated for comparison baseline - month 3 (Table 1, hierarchical listing of tests per domain according to effect sizes). Effect sizes of the high-frequency testing phase (baseline - month 3) are mainly within the range of moderate effects (*d *= 0.51-0.75) and congruent with repeated-measures ANOVA results. A domain-wise comparison of effect sizes demonstrates a homogenous pattern in all cognitive domains (mainly moderate effects) except for attentional functions (high variance with small to large effect sizes).

Upon long intertest intervals after month 3 until study end (month 12) no significant performance changes could be found in 23 of 25 tests, i.e. performance levels acquired by high-frequency testing remained stable and did not return to baseline values. Only one test, RWT phonemic verbal fluency, showed further enhanced test scores (p < 0.001). A ceiling effect in RBANS Picture Naming during high- and low-frequency testing phase artificially produced significant results (Table 1).

**Figure 2:**
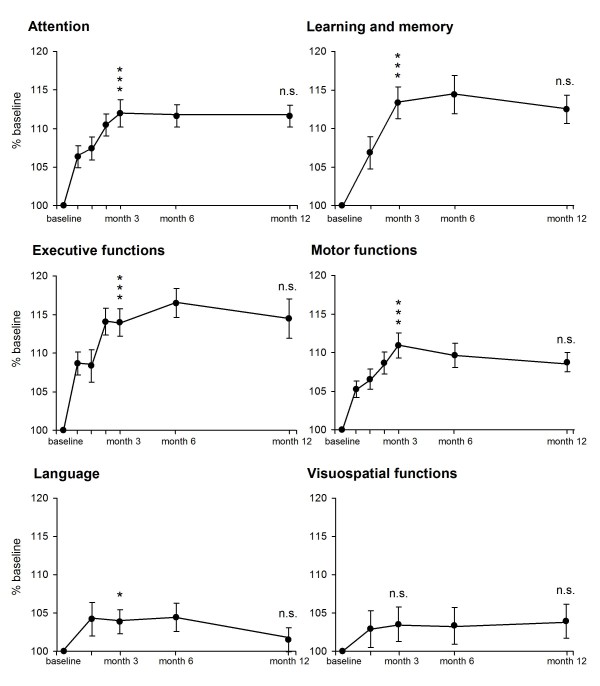
**Pattern of practice effects in cognitive domains over time**. Data of all single tests (always expressed as % individual baseline of the respective test), representing one particular cognitive domain, were combined to yield respective super-ordinate cognitive mean curves. In almost all cognitive domains, changes in total test scores over time exhibit a similar practice pattern: significant improvement during the high-frequency testing phase and stabilization of performance during the low-frequency testing phase. Most pronounced score increases are seen in executive functions as well as in learning and memory, whereas changes in visuospatial performance fail to reach significance. Significance refers to a main effect of time determined with ANOVA for repeated measures, including all testing time-points from baseline to month 3, or from month 3 to month 12, respectively. Mean ± SEM given. ***p < 0.001; *p < 0.05; n.s., not significant.

The longitudinal course of performance in all 6 cognitive domains (data of single tests were combined to yield respective super-ordinate cognitive categories) is illustrated in Figure 2. ANOVAs conducted on this data confirmed the time pattern of single test comparisons: Strong practice effects upon high-frequency testing and a plateau held with decreasing frequency. Regarding cognitive domains, the most pronounced changes occur until month 3 in executive functions (14.0 ± 10.7%), followed by learning/memory (13.3 ± 12.3%) and attention (11.9 ± 10.6%) (Figure 2).

**Figure 3:**
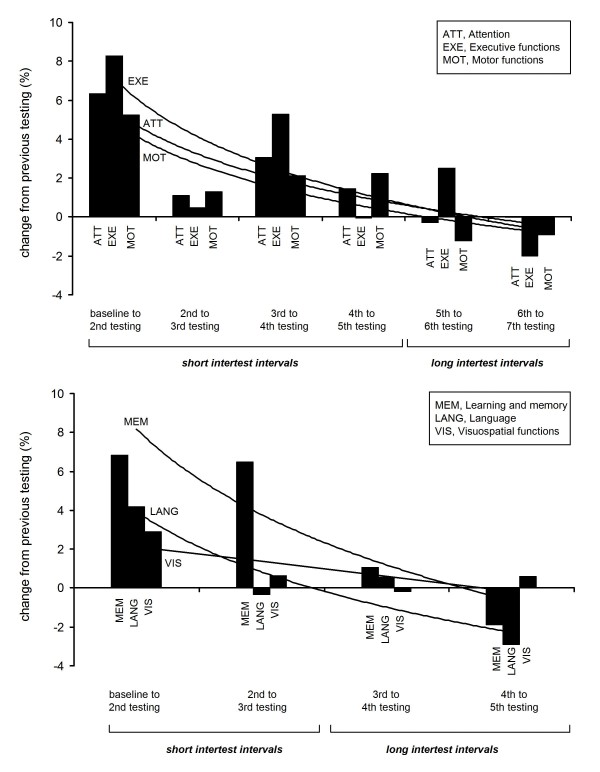
**Distribution of practice effects: Changes from one testing to the next**. All cognitive domains (respective single tests grouped as described in Figure 2) show most pronounced improvement in performance from baseline to the 2^nd ^testing time-point. At late testing time-points with long intertest intervals (5^th ^to 6^th^, and 6^th ^to 7^th ^testing), test scores show a slight tendency to decrease. Mean of % change given. Lines indicate logarithmic trends.

Improvement from baseline to second testing accounts for the largest proportion of change in all cognitive domains (Figure 3). Accordingly, for most tests, Cohen's *d *was highest for baseline - week 2-3 interval (*d *= 0.30-0.55 for domains, *d *= 0.22-0.71 for single tests). In contrast, Cohen's *d*, if calculated for the late between-assessment intervals, i.e. from month 3 to 6 or 12, would show mainly 'no effect' (exceptions: *d *= 0.28 for RWT phonemic verbal fluency and *d *= 0.50 for RBANS Picture Naming).

**Figure 4:**
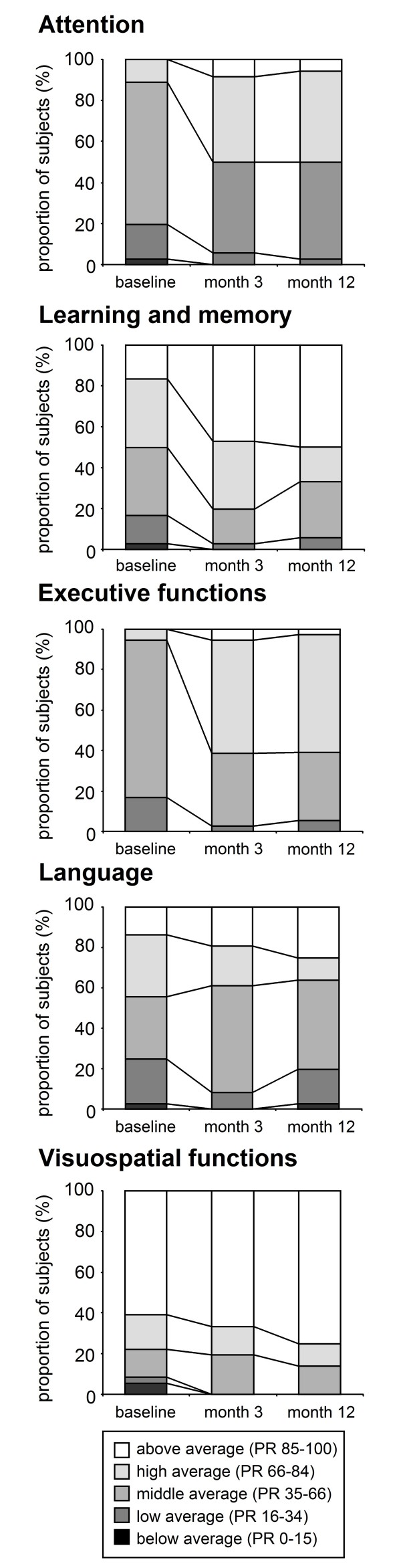
**Magnitude of practice effects: development of clinical classification over 1 year**. Clinical classification of baseline performance shows that cognitive performance is distributed across all categories (below- to above-average percentile ranks, PR) despite a high-IQ sample of healthy individuals. In all depicted cognitive domains, score gains lead to better clinical classification over time (selected time-points months 3 and 12 presented) without reaching upper limits for most subjects. Only in visuospatial functions, the majority of subjects achieved highest scores already at baseline with only modest subsequent changes, altogether pointing to a ceiling effect. Clinical classifications of individual performance are based on test-specific normative data and averaged by cognitive domains. For executive functions, normative data of RWT phonemic verbal fluency is unavailable. Data on motor tests are not presented due to insufficient normative data.

To address the important question of ceiling effects, the proportion of subjects reaching a defined performance level at baseline, month 3 and month 12 was calculated (Figure 4; clinical classifications based on test-specific normative data). For all cognitive domains, changes of clinical classification over time still exclude ceiling effects except for visuospatial functions.

### Control of potentially confounding factors: Pearson's correlations at baseline and ANCOVAs on course of cognitive performance up to month 12

#### Age

Since consistently significant correlations between age and cognitive baseline values in most tests with a speed component were found, age was implemented as a covariate in repeated-measures ANCOVA for all cognitive tests. Only for TAP flexibility, a significant time x age interaction effect could be shown (F = 3.43; p = 0.01), whereas all other learning curves were not influenced by age.

#### Intelligence

Neither MWT-B premorbid IQ nor HAWIE-R full scale IQ correlated systematically with cognitive test scores at baseline (only MWT-B IQ - RWT phonemic verbal fluency: r = -0.47, p = 0.004; HAWIE-R IQ - TMT A: r = -0.42, p = 0.01; HAWIE-R IQ - RBANS Figure Copy: r = 0.57, p < 0.001). Accordingly, IQ did not mediate the course of cognitive performance except for RBANS Figure Copy (time x HAWIE-R IQ interaction F = 5.87; p = 0.001). These findings are most likely due to the high and homogenous IQ of our sample (mean MWT-B IQ: 124.2 ± 12.8, mean HAWIE-R IQ: 127.0 ± 14.1).

#### Personality

Explorative correlational analyses of each NEO-PI-R personality factor with each cognitive test at baseline revealed only isolated significance for Agreeableness - MacQuarrie Tapping and Dotting (r = -.041, p = 0.01 for both) and Conscientiousness - RBANS Story Memory (r = -0.41, p = 0.01). Also, none of the 5 personality factors consistently influenced practice effects (only interaction time x Agreeableness F = 5.17; p = 0.001 for RBANS Coding).

#### Psychopathological symptoms and quality of life

As expected in a sample of healthy volunteers, HAMD and PANSS scores were low and showed small variance ('floor effect' - see description of subjects). Only 3 significant correlations with cognition could be determined at baseline: HAMD - TAP Flexibility (r = 0.47, p = 0.004) HAMD - TMT A (r = 0.53, p = 0.001), PANSS - TMT A (r = 0.42, p = 0.01). Over the study period, HAMD remained unchanged (2.1 ± 2.9 to 2.6 ± 2.8; F = 0.545, p = 0.77), and PANSS scores increased marginally from baseline to month 12 (32.3 ± 3.5 to 33.0 ± 3.5, F = 4.97, p = 0.001) but never reached a pathological level, i.e. is clinically irrelevant. A similar pattern was obtained for QoL and cognition: QoL stayed stable over time and yielded only 2 significant and counter-intuitive correlations with cognitive tests at baseline (QoL - RBANS Digit Span: r = -0.42, p = 0.01, QoL - WMS-III Letter Number Sequencing: r = 0.43, p = 0.01).

Taken together, the evaluation of potential modulators of cognitive performance and practice effects (age, IQ, personality factors, QoL, degree of depression and psychopathology) revealed only isolated findings with single cognitive tests at baseline (20 significant of 275 correlations) or the course of cognitive performance (only 3 significant time x covariate interactions of 200 ANCOVAs). Using a conservative approach of alpha adjustment for multiple testing, these isolated findings even disappear. Thus, none of the analyses (Pearson's correlations, repeated-measures ANCOVAs) suggest that the cognitive performance pattern was due to pre-existing intellectual, personality, sociodemographic or to current psychopathological differences that systematically affected the slope of practice effects. All before mentioned data on cognition is therefore presented without any of the explored covariates.

## Discussion

In the present study, we provide for the first time comprehensive data on clinically relevant practice effects in healthy well-performing subjects over a 1-year period of frequent repetitive testing across 6 distinct cognitive domains. During the initial phase of high-frequency testing for 3 months, strong practice effects occur early on, most prominent in executive functions and learning/memory. After 3 months and upon reduced testing frequency, a stabilization/plateau of the acquired cognitive level until study end is observed. Age, intellectual capacity, personality features, or psychopathological scores have no consistent influence on the course of cognitive performance.

Generally, comparisons between the present and previous studies are confounded by different designs, including the use of diverse cognitive tests, fewer repetitions, and/or varying intertest intervals. The finding that strongest changes in performance occur from baseline to the second testing, however, complies well with a number of similar results on distribution of practice effects [10,12,15,17,18,48]. The extent of practice effects observed here even exceeds effect sizes described by Hausknecht et al [10] (*d *= 0.26) or Bird et al [20], using comparable intervals.

In contrast to previous studies, showing a similar magnitude of practice effects short-term [9,12,48], our longitudinal design addresses particular needs of neuroprotective/neuroregenerative treatment trials, including both, a practice and a retention phase. Just McCaffrey et al [25] had a somewhat related long-term design, but only 4 sessions in total (baseline, week 2, months 3 and 6), with the last testing at month 6, and a much shorter test battery. The essential findings of this study are in agreement with the respective parts of the present work. Another study worth mentioning here, provided useful information about practice-dependent test selection to build on, but used only a high-frequency testing schedule (20 sessions in 4 weeks) without long-term follow-up and without change in testing frequency [49].

Regarding the different cognitive domains, executive functions showed highest score increases over time, followed by learning and memory. For executive functions, results of other studies are contradictory [e.g. [17,20,50]], ranging from no over small to strong practice effects. The strong practice effects in almost all executive functions found here are most likely the result of a higher repetition rate (as compared to [20,50]) or the use of less alternate forms (as compared to [17]). In line with our findings, there is wide agreement that memory functions benefit most from practice [7,25,48,51] and are evident even when alternate test forms are applied [10,14,15,17,52]. Since parallel forms were also administered in the present study, and the respective tests were reduced to 4 repetitions, test sophistication [8] as well as improvement of the underlying functions rather than simple recall effects may have contributed to improved performance.

On the basis of single test characteristics and results over time, no prediction can be made regarding the impact of repetitive testing. Practice effects seem to be unrelated to task complexity or modality. On the other hand, the present work provides more than test-specific information: cognitive domains, assessed with an extensive test battery, covering each domain by several tests, revealed very homogenous effect sizes within one domain, i.e. similar practice effects irrespective of the test used, pointing to genuine change in the underlying target domain (transfer effects). Only within the attention domain, highly varying effect sizes of individual tests may indicate respective test specificity [53], e.g. in our study TAP Visual Scanning displayed largest practice effects whereas RBANS Digit Span revealed no effects. In the overall picture of transfer effects, the few tests with ceiling effects did not play a role in this respect.

Logically, our findings on practice effects raise the question whether after 3 months of regular practice the maximally possible improvement is already achieved or whether continued practice would lead to an even more enhanced performance. Even though this was not the objective of the present study, it would be interesting to investigate how many additional sessions within the high-frequency period are required until the individual upper performance limit is reached.

Although the majority of tests showed considerable practice effects, at least one test in most of the cognitive domains was found resistant to practice. Again, task complexity does not seem to be the underlying factor explaining resistance. For more 'deficit-oriented' subtests like RBANS List Recognition, Lines and Figure Copy, ceiling effects (expected especially in high IQ subjects) did not allow further improvement of test scores. For most other tests this was not the case since the majority of subjects, despite high IQ, did not score at above-average. Nevertheless, the high IQ level of our sample may have contributed to the observed strong practice effects as reported in studies showing that high IQ subjects benefit more from prior test exposure ('the rich get richer' [16,18]). This greater benefit of high IQ, however, is still equivocal as is a potential influence of age [20,50]. In fact, neither age nor IQ, applied as covariates, revealed a clear effect in the present work. Also other covariates, i.e. personality and psychopathology ratings, failed to show any appreciable impact on learning curves. The most plausible explanation would be the fact that healthy volunteers scored in a very restricted 'normal' range in these categories. Such restricted range holds similarly true for IQ.

The aim of the present study, apart from long-term analysis of practice effects, was to provide recommendations for an 'ideal' neuropsychological test battery suitable for serial testing in research and routine. As obvious from our results, two major points have to be considered in this recommendation: test selection and timing. Tests of first choice are those that are essentially resistant to practice: TAP Alertness or RBANS Digit Span for attention; TAP Working Memory for executive functions; MacQuarrie Dotting for motor functions; RBANS Semantic Fluency for language.

For learning and memory, no practice-resistant valid test could be identified. Therefore, for evaluation of this particular domain, a 'dual baseline' approach [5,6] is suggested to partly cut off early practice effects: If the most prominent improvement occurs from first to second assessment, the second may serve as baseline for subsequent assessments. For the domain learning and memory, this applies to RBANS Figure Recall, List Recall and List Learning.

As eventual alternatives for the above listed domain-specific, practice-resistant tests, the dual baseline approach may be used for TMT A, RBANS Coding (attention), WCST-64, WMS-III Letter Number Sequencing, RWT phonemic verbal fluency (executive functions) and MacQuarrie Tapping (motor functions). Of all the explored cognitive domains, only for visuospatial functions a valid test recommendation cannot be made at this point.

The selection of tests for a neuropsychological battery is often a matter of compromises and limitations. Due to time restrictions and fatiguing effects, it is impossible to completely cover all relevant cognitive domains with all their facets in one session. For instance, in this comprehensive test battery, data on inhibition control or interference resolution as important aspects of executive function had to be omitted due to these restrictions. On the other hand, some deficit-oriented tests, essential for clinical studies, were selected that ultimately displayed ceiling effects in the healthy sample. Especially for the domains visuospatial functions and language, not only more tests but also more suitable tests have to be identified and investigated longitudinally.

In addition to our recommendations for an optimal, practice-resistant test battery, also our data of tests with strongest practice effects are useful for future applications. Based on reliable change index calculations, hierarchical linear modelling or regression models, it will now be possible to discriminate whether performance change of an individual or a group is clinically meaningful or whether it simply reflects change due to the here described practice effects.

## Conclusions

Although the present study with its asymmetrical testing design addresses particularly needs of neuroprotective trials, the principal findings on practice effects also apply to all kinds of clinical and non-clinical studies with repetitive short- and long-term neuropsychological testing. Based on the here reported results, an essentially complete cognitive test battery covering all major cognitive domains can be composed. This battery should be largely resistant to practice or at least allow a valid estimate of practice effects. Thus, true cognitive improvement will be better discernible in healthy individuals and even more so in patient populations with expectedly reduced capabilities to learn [31,49,54,55]. Along these lines, the collection of normative data for serial test administration as important information on individual longitudinal learning can now easily be initiated.

## Authors' contributions

All authors qualify for authorship, based on their substantive intellectual contributions to the study. HE and CB designed the present study and wrote the manuscript. CB, MW, AW and VA participated in acquisition and analysis of the presented data. MW, AW and VA also contributed to the interpretation of data and helped revising the manuscript critically for important intellectual content. All authors gave final approval of the version to be published.

## Supplementary Material

Additional file 1 **Detailed descriptive information on the neuropsychological test battery**. This file contains descriptive information on the neuropsychological tests of the presented study (underlying function, procedure) and an overview of alternate test versions. Click here for file
