# Supplementary material for: Practice effects in healthy adults: A longitudinal study on frequent repetitive cognitive testing
Source: BMC Neurosci. 2010 Sep 16;11:118. doi: 10.1186/1471-2202-11-118 (PMC2955045; doi:10.1186/1471-2202-11-118)
Supplement: Additional file 1 — Detailed descriptive information on the neuropsychological test battery. This file contains descriptive information on the neuropsychological tests of the presented study (underlying function, procedure) and an overview of alternate test versions. [file 1471-2202-11-118-S1.DOC]

**Additional File 1**

**for**

**Practice effects in healthy adults:**

***A longitudinal study on frequent repetitive cognitive testing***

by

Claudia Bartels* PhD, Martin Wegrzyn BS, Anne Wiedl MS, Verena Ackermann BS,

and Hannelore Ehrenreich* MD DVM

**Detailed descriptive information on the neuropsychological test battery**

***Attention***

Trail Making – part A (TMT A; [1]) is a simple task focusing on visuomotor processing speed. Subjects are asked to connect numbers up to 25 consecutively on a work sheet as fast as they can.

Repeatable Battery for the Assessment of Neuropsychological Status (RBANS; [2]) is a brief testing battery, originally designed for cognitive screening of elderly subjects with suspected dementia, but also serves as general test battery. A total of 12 subtests are subsumed under 5 index scores (mean of 100±15). Additionally, a summary score can be determined. The subtest Coding (Attention Index) primarily assesses complex scanning and visual tracking. On a work sheet, pairs of symbols and digits from 1 to 9 are presented, followed by rows of symbols without the respective digit. Within a time limit of 90 seconds, test subjects are asked to assign as many numbers to symbols as they can.

RBANS subtest Digit Span (Attention Index): In this simple task, involving auditory attention and short-term retention capacity of the phonological loop, random number sequences are read aloud to the test subject and have to be repeated immediately. Number sequences increase in complexity, ranging from 2 to 9 digits.

Test for Attentional Performance (TAP; [3]) subtest Visual Scanning: This test assesses tracking of the visual field and, due to its amount of trials (100 in total), sustained attention. In a matrix-like presentation of 5x5 stimuli, subjects have to detect if a pre-specified critical stimulus is shown or not. After a response (“present” or “not present”) on the respective reaction key, the next matrix appears.

TAP subtest Alertness measures the subject’s ability to respond to a visual stimulus and to increase the attentional level in expectance of a stimulus of high priority. The computer-assisted test is given under 2 conditions: (1) Simple reaction time to a visual stimulus (Greek cross) appearing at randomly varying intervals on the monitor screen is measured. (2) In the second condition, the visual stimulus (= critical stimulus) is preceded by a cue sound presented as warning tone.

***Learning and memory***

RBANS subtest Figure Recall (Delayed Memory Index): In order to assess visual long-term memory, the complex figure that has to be copied at the beginning of the RBANS (see also Visuospatial functions, RBANS subtest Figure Copy), has to be recalled after an interval of approximately 20 minutes.

RBANS subtest List Learning (Immediate Memory Index): A non-organized list of 10 words is used to measure verbal learning and short-term memory. The orally presented word list has to be recalled immediately, followed immediately by 3 more trials in which the same word list is repeated and recalled each time.

RBANS subtest List Recall (Delayed Memory Index): In a free recall modality, the same word list has to be recalled after a delay of approximately 20 minutes, indicating verbal long-term memory functions. Afterwards, in the RBANS subtest List Recognition (Delayed Memory Index) the 10 correct words, mixed with 10 distractor items, have to be identified.

RBANS subtest Story Memory (Immediate Memory Index): Also measuring verbal short-term memory, but in this case using structured material, this subtest requires immediate recall of a short prose passage (2 trials).

RBANS subtest Story Recall (Delayed Memory Index): Similar to the subtest List Recall, this task aims at assessing verbal long-term functions, demanding free recall of the story presented approximately 20 minutes before.

***Executive functions***

Trail Making - part B (TMT B; [1]): Additionally to the visuomotor processing speed component of part A, part B also requires divided attention and cognitive flexibility. In this speed test, subjects have to connect consecutively numbers (up to 13) and letters (from A to L) in an alternating sequence (set shifting).

Wisconsin Card Sorting Test – 64 Card Version (WCST-64; [4]): This test of concept formation and cognitive flexibility is widely used as one of the standard tests for frontal lobe dysfunction. From a card desk of 64 cards, subjects have to assign single cards to one of 4 stimulus cards (differing in color, form and quantity). The subject has to find the correct concept by reacting to the examiner’s response (“correct” or “incorrect”). After 10 correct responses in a row, the category changes (complete category sequence: color-form-number-color-form-number). The complexity of the test is caused by the test taker’s unawareness of the category sequence and the time-point of change.

TAP subtest Flexibility (condition “verbal”, complex mode) is a set shifting task. It starts with the presentation of a letter and a number positioned at the right and left centre of the monitor. The subject should first press a key on the side where the letter is presented. On the next screen with a new digit and letter, the subject has to respond to the digit. In this alternating fashion, the procedure goes on for 100 trials in total.

TAP subtest Working Memory mainly focuses on the control of information flow and information updating, the major working memory task. By a key press, the test-taker has to determine whether a presented digit corresponds to the one before that.

Wechsler Memory Scale – 3^rd^ edition (WMS-III; [5]) subtest Letter Number Sequencing: This working memory test makes high demands on the central executive component of working memory. Mixed sequences of digits and letters are presented loudly and have to be recalled in an ascending order with all digits first, followed by all letters. The task comprises sequences with a length of 2 to 8 items.

Regensburger Wortschatztest (RWT; [6]) subtest phonemic verbal fluency is also known as letter fluency. It consists of oral production of spoken words within 1 minute beginning with a certain letter (e.g. “S”). To perform the complete subtest, 4 letters with different search spaces are given. A number of restrictions (e.g. no proper nouns, no repetitions) set limits to the search space.

***Motor functions***

The 9-Hole Peg Test [7] is part of the Multiple Sclerosis Functional Composite (MSFC) and comprises a method for measuring upper extremity (fine) motor function. 9 pegs – one at a time – have to be put into 9 holes of a plastic block and afterwards to be removed as quickly as possible. Dominant and non-dominant hands are tested twice and averaged or the best performance is counted.

Purdue Pegboard Test [8]: This manual dexterity test includes 4 different conditions: (1) Pegs are first placed with the dominant, (2) then the other hand, (3) and then with both hands simultaneously (30 seconds per trial). In the fourth condition, different pegs are assembled to create a small ‘tower’, using both hands alternately (1 minute). Each condition is tested once.

MacQuarrie Test for Mechanical Ability subtests Tapping and Dotting [9]: While Dotting requires fine motor accuracy to place one dot each in a row of small circles, Tapping only measures visuomotor speed. In the latter test, subjects are asked to make 3 pencil dots each in a series of circles. For both tests, the time limit is 30 seconds in which as many dots as possible have to be made.

***Language***

RBANS subtest Picture Naming (Language Index) is a simple task of naming 10 familiar objects used for the assessment of basic verbal functions and language skills. If the subject is unable to give the correct name, cues are allowed.

RBANS subtest Semantic Fluency (Language Index) refers to a category fluency task. Within 1 minute, as many words of a restricted category (fruits/vegetables or zoo animals) as possible have to be generated.

***Visuospatial functions***

RBANS subtest Lines (Visuospatial/Constructional Index) can be seen as a short and modified version of Benton’s Judgment of Line Orientation. The test consists of 10 items, each showing a pair of angled lines to be matched to the display of 13 lines forming a semicircle at the top of the work sheet.

RBANS subtest Figure Copy (Visuospatial/Constructional Index): Performance on this subtest is mainly based on perceptual organization. Test-takers are instructed to accurately copy a complex figure. Time for copying is limited to 4 minutes.

***Intelligence***

Mehrfachwahl-Wortschatz-Intelligenz-Test (MWT-B; [10]) is a measure of premorbid / crystallized intelligence. Using multiple choice, one word out of 4 nonsense words has to be identified. The test includes 37 items with increasing complexity.

Hamburg-Wechsler-Intelligenz Test für Erwachsene – Revision (HAWIE-R; revised German version of the Wechsler Adult Intelligence Scale, short version; [11]): A short version, comprising the subtests Information and Similarities, Picture Completion and Block Design, was used to determine full scale, verbal and performance intelligence.

**Supplementary Table 1: Overview of alternate test versions over all test sessions**

|  |  |  |  | **Version used** | | |  |  |  |
| --- | --- | --- | --- | --- | --- | --- | --- | --- | --- |
| **Test parameter** | **Baseline** | **Week 2-3** | **Week 6** | | **Week 9** | **Month 3** | | **Month 6** | **Month 12** |
| ***Attention*** |  |  |  | |  |  | |  |  |
| TAP Visual Scanning | n.a. | n.a. | n.a. | | n.a. | n.a. | | n.a. | n.a. |
| Trail Making Test – part A | 1 | 1 | 1 | | 1 | 1 | | 1 | 1 |
| RBANS Coding | A | A | B | | B | A | | B | A |
| TAP Alertness | n.a. | n.a. | n.a. | | n.a. | n.a. | | n.a. | n.a. |
| RBANS Digit Span | A | A | B | | B | A | | B | A |
| ***Learning and memory*** |  |  |  | |  |  | |  |  |
| RBANS Figure Recall | A | - | B | | - | A | | B | A |
| RBANS List Recall | A | - | B | | - | A | | B | A |
| RBANS List Learning | A | - | B | | - | A | | B | A |
| RBANS Story Memory | A | - | B | | - | A | | B | A |
| RBANS Story Recall | A | - | B | | - | A | | B | A |
| RBANS List Recognition | A | - | B | | - | A | | B | A |
| ***Executive functions*** |  |  |  | |  |  | |  |  |
| Trail Making Test – part B | 1 | 1 | 1 | | 1 | 1 | | 1 | 1 |
| WCST-64 | n.a. | n.a. | n.a. | | n.a. | n.a. | | n.a. | n.a. |
| TAP Flexibility | n.a. | n.a. | n.a. | | n.a. | n.a. | | n.a. | n.a. |
| WMS-III Letter Number Sequencing | n.a. | n.a. | n.a. | | n.a. | n.a. | | n.a. | n.a. |
| RWT phonemic verbal fluency | 1 | 2 | 3 | | 4 | 1 | | 2 | 1 |
| TAP Working Memory | n.a. | n.a. | n.a. | | n.a. | n.a. | | n.a. | n.a. |
| ***Motor functions*** |  |  |  | |  |  | |  |  |
| 9-Hole Peg Test | n.a. | n.a. | n.a. | | n.a. | n.a. | | n.a. | n.a. |
| Purdue Pegboard Test | n.a. | n.a. | n.a. | | n.a. | n.a. | | n.a. | n.a. |
| MacQuarrie Tapping | n.a. | n.a. | n.a. | | n.a. | n.a. | | n.a. | n.a. |
| MacQuarrie Dotting | n.a. | n.a. | n.a. | | n.a. | n.a. | | n.a. | n.a. |
| ***Language*** |  |  |  | |  |  | |  |  |
| RBANS Picture Naming | A | - | B | | - | A | | B | A |
| RBANS Semantic Fluency | A | - | B | | - | A | | B | A |
| ***Visuospatial functions*** |  |  |  | |  |  | |  |  |
| RBANS Lines | A | - | B | | - | A | | B | A |
| RBANS Figure Copy | A | - | B | | - | A | | B | A |

TAP, Test for Attentional Performance; RBANS, Repeatable Battery for the Assessment of Neuropsychological Status; WCST-64, Wisconsin Card Sorting Test – 64 Card Version; WMS-III, Wechsler Memory Scale – 3^rd^ edition; RWT, Regensburger Wortflüssigkeitstest; n.a., not available.

**Test references**

1. Reitan RM: **Validity of the Trail Making Test as an indicator of organic brain damage.** *Percept Mot Skills* 1958, **8:**271-276.

2. Randolph C: *RBANS Manual - Repeatable Battery for the Assessment of Neuropsychological Status.* San Antonio, TX: The Psychological Corporation (Harcourt); 1998.

3. Zimmermann P, Fimm B: *Test for Attentional Performance (TAP; German:Testbatterie zur Aufmerksamkeitsprüfung).* Herzogenrath: Psytest; 1995.

4. Kongs SK, Thompson LL, Iverson GL, Heaton RK: *WCST-64: Wisconsin Card Sorting Test-64 Card Version; Professional Manual.* Odessa, FL: Psychological Assessment Resources, Inc.; 2000.

5. Wechsler D: *Wechsler Memory Scale - 3rd edition (WMS-III).* Harcourt, TX: The Psychological Corporation; 1998.

6. Aschenbrenner S, Tucha O, Lange KW: *Regensburger Verbal Fluency Test (RWT; German: Regensburger Wortflüssigkeits-Test).* Göttingen: Hogrefe; 2000.

7. Cutter GR, Baier ML, Rudick RA, Cookfair DL, Fischer JS, Petkau J, Syndulko K, Weinshenker BG, Antel JP, Confavreux C: **Development of a multiple sclerosis functional composite as a clinical trial outcome measure.** *Brain* 1999, **122:**871-882.

8. Tiffin J: *Purdue Pegboard examiner manual* Chicago, IL: Science Research Associates; 1968.

9. MacQuarrie TW: *MacQuarrie Test for Mechanical Ability.* Monterey, CA: California Test Bureau/McGraw-Hill; 1925, 1953.

10. Lehrl S: *Multiple Choice Vocabulary Intelligence Test (MWT-B; German: Mehrfachwahl-Wortschatz-Intelligenztest).* Erlangen: Straube; 1995.

11. Tewes U: *German Adaption of the Revised Wechsler Adult Intelligence Scale (HAWIE-R; German: Hamburg-Wechsler Intelligenztest für Erwachsene - Revision* Bern: Hans Huber; 1991.
